# Editorial: Plant Pathogen Life-History Traits and Adaptation to Environmental Constraints

**DOI:** 10.3389/fpls.2019.01730

**Published:** 2020-01-24

**Authors:** Christophe Le May, Josselin Montarry, Cindy E. Morris, Omer Frenkel, Virginie Ravigné

**Affiliations:** ^1^ IGEPP, INRA, Agrocampus-Ouest, Université de Rennes 1, Le Rheu, France; ^2^ INRA, UR Pathologie Végétale, Avignon, France; ^3^ Agricultural Research Organization, Volcani Center, Rishon LeZion, Israel; ^4^ BIOS Departement, PVBMT Laboratory, CIRAD, Montpellier, France

**Keywords:** coinfection, epidemic, host resistance, hydric stress, leaf maturity, non-agricultural habitats, survival, temperature

Life-history is a key concept in evolutionary biology and ecology. It corresponds to the narrative of the various events punctuating the existence of an organism from his birth to his death (Begon et al., 2006; Michalakis et al., 2016). Throughout life, living organisms acquire resources that they actively find or extract from their environment and then allocate to different functions: development, survival, dispersal and reproduction (Roff, 1992; Stearns, 1992; Michalakis, 2009). Traits involved in the timing and amplitude of these allocation dilemmas are defined as life history traits. Life-history traits are often determinant for individuals (Kingsolver and Pfennig, 2007). They influence spatial and temporal disease dynamics, and thus the genetic diversity and structure of pathogen populations (Barrett et al., 2008; Michalakis et al., 2016). These determinants are involved in the ability of pathogens to adapt to varying ecological factors including changes in the biotic and abiotic effect, but also direct or indirect interactions with other strains or species of pathogens co-occurring on the same host (Michalakis et al., 2016; Tollenaere et al., 2016). For plant pathogens, these traits can be grouped into two categories: those involved in the epidemic phase, onto or into the host, and those related to the survival phase, often outside the host. Trade-off can occurred between these two phases, leading to consequences on epidemic dynamics, evolution, and speciation (Pariaud et al., 2009; Hamelin et al., 2011). Understanding processes maintaining variation in plant pathogen life-history traits is a central question in evolutionary ecology and a major challenge for the design of disease control strategies (Galvani, 2003; Grenfell et al., 2004).

The aim of this Research Topic was to collect research papers with vast perspective on plant pathogen life-history traits and adaptation to environmental constraints. Life-history traits of both epidemic and survival phases were considered, and tested environmental constraints included abiotic (temperature, precipitation, hydric stress) and biotic (habitat of origin, cultivar, maturity of leaves, coinfection) factors. Among the 10 accepted papers, three types of plant pathogens were considered: fungi (seven papers), nematodes (two papers), and oomycetes (one paper).

Regarding the epidemic phase, four main topics were covered by the research papers accepted in this special issue: *i)* host adaptation, *ii)* impact of host’s physiological status, *iii)* adaptation to climatic conditions, and *iv)* effects of coinfection.

How host adaptation affects life history traits evolution was particularly analyzed by studying the effect of resistant cultivars onto several life-history traits. In the ascomycete *Colletotrichum gloeosporioides* the impact of host quantitative resistance on pathogen evolution was studied by evaluating the diversity of populations at the field scale at both neutral markers and pathogenic traits (Frezal et al.). Population genetic structure revealed a significant influence of clonal reproduction in *C. gloeosporioides* evolution and a low migration rate between fields. Results of cross-inoculation tests showed that aggressiveness of the fungal clones seemed to have evolved through an accumulation of components specific to each water yam cultivar, suggesting an adaptation to their host cultivar. Despite the remaining marks of adaptation to the former widely cultivated host, adaptation to current cultivars was clearly depicted. Such pattern of local adaptation to the dominant cultivar was previously highlighted in the potato late blight pathogen *Phytophthora infestans* (Andrivon et al., 2007). In their study, Mariette et al. explored whether the now classical negative relationship between offspring size and number occurred in this oomycete, *P. infestans*, and whether the trade-off was impacted by potato cultivar or host of origin of the pathogen (tomato and potato). They confirmed the existence of a trade-off and showed that it was not affected by any of these biotic factors. The observed polyphenism for these traits in *P. infestans* populations will favor the coexistence of distinct reproductive strategies.

Over the last years, intra-host dynamics has been identified as a major component of epidemiological and evolutionary dynamics of pathogens (Alizon et al., 2009). In plants it is known that host tissue characteristics vary a lot with tissue age. In particular, according the age of tissues, nutrient availability as well as defense levels may vary significantly, with opposing effects on the success of infection (Kus et al., 2002; Al-Naimi et al., 2005). Ontogenic resistance against powdery mildew (*Erysiphe necator)* on *Vitis vinifera* was studied by Calonnec et al. They showed that the three pathogenic traits studied (infection efficiency, sporulation, and mycelium growth) were affected, and their variation was strongly correlated with leaf age. Sporulation was more closely correlated with variations in sugar contents and the infection efficiency with leaf water content, suggesting that ontogenic resistance on grapevine leaves seems to be an immutable physiological process that *E. necator* is able to circumvent by restricting its development to sink tissue. Similarly, Maupetit et al. tested how nutrient availability and defense level impacted the development of *Melampsora larici-populina* (infection efficiency, latent period, uredinia size, mycelium quantity, sporulation rate, sporulation capacity, and spore volume). They showed that *M. larici-populina* was more aggressive on more mature leaves as indicated by wider uredinia and a higher sporulation rate. In contrast, phenolic contents (flavonols, hydroxycinnamic acid esters, and salicinoids) were negatively correlated with uredinia size and sporulation rate suggesting that pathogen’s fitness appeared to be more constrained by the constitutive plant defense level than limited by nutrient availability, as evident in the decrease in sporulation.

Like other organisms, pathogens need a particular range of temperature and humidity in order to invade and develop on their host plant (Agrios, 2005). These two parameters particularly can modify spore germination, host colonization, spore production and dispersal of fungal pathogens (Fitt et al., 2006). Decades of research have generated considerable knowledge and greater understanding of the seasonal effects of temperature, rainfall, and humidity on diseases affecting major food crops (Garrett et al., 2011; Pautasso et al., 2011; Donatelli et al., 2017). In this special issue, two main parameters were studied, change in temperature and effect of water stress. Regarding the effect of temperature, Vaumourin and Laine developed a study on *Podosphaera plantaginis* and showed that temperature had a significant effect on all measured life-history traits, suggesting that the effect of temperature on life-history traits was both direct as well as mediated through a genotype-by-temperature interaction. Similarly, Mariette et al. showed on *P. infestans* that the negative relationship between offspring size and number was maintained for all the tested temperatures. Using cyst nematodes, other studies showed an effect of temperature on different life-history traits. Indeed, Fournet et al. showed a strong temperature effect on life-history traits of the beet cyst nematode *Heterodera schachtti*. While nematode multiplication was not differentially affected by temperatures, as favorable conditions for the host are also favorable for the parasite, the effect of temperature on hatching depended on the origin of populations, separating southern from northern European populations. Using a genome scan approach, Gendron St-Marseille et al. evaluated the adaptive potential of the soybean cyst nematode *Heterodera glycines*. The genotyping by sequencing of 64 *H. glycines* populations, allowed identifying 15 loci under selection for climatic or geographic co-variables. Lastly, regarding the effect of water stress, a transcriptomic and metabolomic approach was developed on the *Arabidopsis thaliana/Alternaria brassicicola* pathosystem, to study the level of susceptibility of the fungus to water stress and its impact on its seed transmission ability (N’Guyen et al.). These approaches led to the identification of specific proteins (hydrolipin) implicated in the tolerance toward water stress, and seed transmission, but not in the aggressiveness of the pathogen.

The ability of a pathogen to establish and grow on its host may be drastically altered by simultaneous infection by other pathogens (other strains or species of pathogens co-occurring on the same host). Life-history allocation may change under coinfection, affecting the evolutionary potential and epidemiological dynamics of pathogens (Tollenaere et al., 2016; Laine and Mäkinen, 2018). In their study, Vaumourin and Laine found that coinfection only modified the number of sexual resting structures produced, but did not changed the production of asexual forms. Coinfection studies, assessment of fungi sharing common characteristics, and host species, create a challenge for conventional disease diagnosis and subsequent management strategies. Indeed, lack of powerful tools to simultaneously distinguish and quantify these types of pathogens constitutes a major limitation. In their study, Abdullah et al. developed a duplex real-time PCR assay for quantifying of co-infecting wheat pathogens, *Pyrenophora tritici-repentis* and *Parastagonospora nodorum.* The utility of the method was demonstrated using field samples of a cultivar sensitive to both pathogens. While visual and culture diagnosis suggested the presence of only one of the pathogen species, the assay revealed not only presence of both co-infecting pathogens (hence enabling asymptomatic detection) but also allowed quantification of relative abundances of the pathogens as a function of disease severity.

Survival stage outside the host has often been much overlooked due to the difficulty of its study. It is a key stage in pathogen’s life cycle. Pathogens are all dependent on the life cycle, where despite the existence of dormant forms, evolutionary processes may occur. Thus, according to the status of their host (perennial or annual host plant), pathogens have to develop a wide array of strategies to overcome the alternation of presence/absence (and/or modification of physiological activity) of their host (Agrios, 2005). This survival phase is consequently important as it may affect the population size of the pathogen and lead to a bottleneck process. In this, special issue, research developed by Bardin et al. have investigated the ubiquity of the broad host range necrotrophic fungus *Botrytis cinerea*, outside of agricultural settings and have determined whether the populations in these natural habitats can be distinguished phenotypically and phylogenetically from populations isolated from diseased crops. Their results showed that *B. cinerea* strains sampled on different non-agricultural substrates were genetically and phenotypically similar to strains sampled in agricultural substrates. These results suggest that highly diverse populations of this plant pathogen persist outside of agriculture in association with substrates other than cultivated plants and that this component of their life-history is compatible with its capability to maintain its potential as plant pathogen. The survival phase was also considered in Vaumourin and Laine, as coinfection affects the number of sexual resting structures produced, and in Fournet et al., as they considered also the hatching after storage at different temperatures, simulating survival conditions during the inter-cropping period.

Considering plant pathogen traits in the context of life history trait evolution is a promising research avenue, still in its infancy. We hope that this research topic will help foster this line of research and provide a valuable resource for researchers working in the field of evolutionary ecology of plant pathogen populations.

## Author Contributions

CL, JM, CM, OF, and VR have made substantial direct contributions to the work, and approved it for publication.

## Conflict of Interest

The authors declare that the research was conducted in the absence of any commercial or financial relationships that could be construed as a potential conflict of interest.
